# Associations Between Secondhand Smoke Exposure During Pregnancy and Preterm Birth

**DOI:** 10.3390/jcm14124325

**Published:** 2025-06-17

**Authors:** Arwa A. Al-Mughathwai, Mai Alharbi, Tahani Aljehani, Bushra Alsamayil, Rehab Alruwathi, Amal Zaman, Samah Alfahl, Abdulmohsen H. Al-Zalabani

**Affiliations:** 1 Joint Program of Preventive Medicine, Taibah University, Madinah 42353, Saudi Arabia; malharbi01@taibahu.edu.sa; 2 King Salman Medical City, Madinah Maternity Children Hospital, Madinah 42319, Saudi Arabiabalsamayil@moh.gov.sa (B.A.); ralruwathi@moh.gov.sa (R.A.); 3 Department of Obstetrics and Gynecology, Taibah University, Madinah 42353, Saudi Arabia; ayzaman@taibahu.edu.sa; 4 Department of Family and Community Medicine, Collage of Medicine, Taibah University, Madinah 42353, Saudi Arabia; sfahl@taibahu.edu.sa (S.A.); azalabani@taibahu.edu.sa (A.H.A.-Z.)

**Keywords:** secondhand smoking, preterm birth, pregnancy, Saudi Arabia

## Abstract

**Background:** Exposure to secondhand smoke (SHS) during pregnancy has been linked to adverse maternal and perinatal outcomes. Preterm birth is one of the most critical complications. However, the evidence of an association between SHS exposure and preterm birth remains inconclusive. **Objectives:** our aim was to identify the prevalence of exposure to secondhand smoke among pregnant women and investigate the association between exposure to SHS and preterm birth. **Methods**: A cross-sectional study was conducted in Madinah, Saudi Arabia, from October to December 2024. A self-administered questionnaire was distributed among women in the postnatal ward to assess sociodemographic and pregnancy-related variables and exposure to SHS. **Results**: A total of 469 women were included in the study. About 33.7% of the women reported that their husbands currently smoked tobacco, with 51% smoking indoors; 21% of the women did not impose smoking restrictions at home. Pregnant women exposed to SHS had more than twice the odds of preterm birth compared to unexposed women (OR = 2.09; 95% CI: 1.06–4.13; *p* = 0.03). **Conclusions:** SHS exposure increased the risk of preterm birth among pregnant women in Madinah. These findings emphasize the essential need for preventive strategies to diminish SHS exposure in residential and public environments.

## 1. Introduction

Smoking constitutes a major global public health concern as a substantial risk factor for morbidity and mortality [1]. The health risks of smoking are not limited only to smokers, as they also have an impact on exposed non-smokers [2]. Secondhand smoke (SHS) refers to smoke that is released from a cigarette or another form of tobacco product and inhaled by someone other than the smoker. SHS is also called passive or environmental smoke [3]. According to the World Health Organization, around 1.2 million fatalities each year are attributed to SHS exposure [4]. SHS involves exposure to over 7000 chemicals, at least several hundred of which are toxic. There is no safe threshold for smoke exposure; even minor contact can be detrimental [5].

Pregnant women and their developing fetuses are a vulnerable group that faces significant risks. Indeed, the negative effects of SHS in pregnancy go beyond maternal health, potentially affecting fetal development and leading to adverse perinatal outcomes. Toxic chemicals in SHS, such as nicotine and carbon monoxide, can pass through the placenta and harm the growing fetus [6]. One of the most critical perinatal outcomes linked to SHS exposure is preterm birth, defined by the World Health Organization (WHO) as “a delivery that occurs before 37 weeks of pregnancy” [7]. Preterm birth is a contributor to neonatal morbidity and premature mortality on a global scale, and is the primary cause of mortality in children below five years. Meanwhile, it can also result in numerous long-term complications, including respiratory, gastrointestinal, and neurodevelopmental disorders [8].

Saudi Arabia has high rates of smoking, especially among men. This poses a unique set of challenges for maternal health due to women’s exposure to SHS within their own households [9]. In a recent cross-sectional study in Madinah, Saudi Arabia, a notably high prevalence of passive smoking among pregnant women was documented, estimated at approximately 46% [10]. Despite national campaigns against smoking and increased awareness about the associated dangers, cultural factors continue to be strong drivers for smoking behavior, especially for individuals in more traditional household arrangements where family members might smoke indoors [11].

While the risks of active smoking among pregnant women are well established, evidence regarding the association between SHS and preterm birth is not conclusive. Evidence from systematic reviews and meta-analyses suggests that pregnant women exposed to SHS may have an increased risk of preterm birth. However, this increased risk was observed in those exposed at home, but not in the workplace or other settings [12]. Geographical variation was also a factor, with statistically significant associations observed in studies conducted in Asia, but not in other regions. There is a scarcity of studies from Saudi Arabia regarding the association.

Therefore, our study aimed to investigate the association between maternal secondhand smoking status and preterm labor in Al Madinah Al Munawwarah, Saudi Arabia, in 2024.

## 2. Materials and Methods

Setting

This cross-sectional analytic study was conducted at the postnatal ward of the obstetric and gynecological department of the Maternity and Children Hospital, a tertiary hospital in Al-Madinah, in the western region of the Kingdom of Saudi Arabia.

Participants

The study included all mothers admitted to the postnatal ward who gave birth to a live singleton infant between October and December 2024. We excluded women who were active smokers, those with multiple pregnancies (twins), or those with newborns with congenital diseases.

Outcome Variable

The main outcome measured in our study was the preterm birth of newborns, defined as delivery before 37 weeks of gestation, according to the WHO definition [7].

Independent Variable

The primary independent variable examined was exposure to SHS. This was defined as exposure to tobacco products such as cigarettes or shisha in the home or an enclosed area in the workplace.

Sampling and Sample Size

Consecutive sampling was performed for all women admitted to the postnatal ward who met the inclusion criteria. A trained team member collected the data. Women were approached during the hospitalized postpartum period in the postnatal ward across different shifts, including morning, afternoon, and midnight, until the desired sample size was reached. We calculated the required sample size according to a 95% confidence level (α = 5%), a margin of error of 5%, and an anticipated frequency of 50%. The minimal sample size was determined to be 383. We maximized the sample size to account for the non-response rate, resulting in a final sample of 469 mothers.

Pilot study

A sample of 30 mothers was selected for a pilot study to check feasibility, test the questionnaire, assess the work of the data collector, and address any problems encountered.

Measurements

Data were collected through the distribution of a validated, structured, self-administered questionnaire (online form). The Arabic version of the questionnaire was assessed by obstetrician consultants and tobacco control experts to ensure face validity. The questionnaire consisted of three main sections: sociodemographics, pregnancy-related information, and tobacco exposure.

The first section included sociodemographic characteristics such as age, education level (illiterate, school education, higher education), employment status (student, housewife, employed), income level (less than 5000 SAR, 5000–10,000 SAR, 10,000–20,000 SAR, more than 20,000 SAR), and presence of any known chronic diseases.

The second section addressed pregnancy-related variables, including mode of delivery, gestational age at delivery (preterm, full-term, or post-date), inter-pregnancy interval, and history of previous intrauterine fetal death.

The third section focused on smoking exposure. SHS exposure was assessed by asking whether the husband or other family members smoked at home, the type of tobacco products used, exposure to SHS in the workplace, and the frequency of such exposure (daily, weekly, or monthly).

Statistical analysis

All statistical analyses were conducted using IBM SPSS Statistics for Windows, Version 26 (IBM Corp., Armonk, NY, USA). A *p*-value < 0.05 was considered statistically significant. Descriptive statistics were used to summarize the demographic, clinical, and behavioral characteristics of the study population. Continuous variables were expressed as the mean ± standard deviation (SD). Categorical variables were summarized using frequencies and percentages.

The association between categorical variables and preterm delivery (Yes, No) was assessed using the chi-squared (χ^2^) or Fisher’s exact test, as appropriate. These analyses were performed for maternal demographic characteristics (e.g., age, education, employment status, income), clinical factors (e.g., previous intrauterine fetal death, chronic disease), and smoking exposure (e.g., SHS exposure, household smoking rules).

To identify independent predictors of preterm birth, a binary multivariable logistic regression model was constructed. Variables with a *p*-value < 0.20 in bivariate analysis were considered for inclusion in the multivariable model. Adjusted odds ratios (ORs) with 95% confidence intervals (CIs) were reported.

Ethical consideration

Ethical approval of the research was obtained from the Research Ethics Committee at King Salman bin Abdulaziz Medical City (IRB log No: 047-24). All participants provided informed consent. Participants were supplied with information on the research’s goal, benefits, and lack of risk; data were guaranteed to be used solely for research purposes while upholding confidentiality and anonymity. Participants had the right to withdraw at any point during the interview.

## 3. Results

A total of 469 women were included in the study. Of these, 28% (*n* = 104) reported exposure to tobacco smoke during pregnancy.

Table 1 shows the sociodemographic distribution of the study population and their past maternal characteristics. In our sample, 16% of the participants were less than 25 years old, 56% were aged 26 to 35 years old, and 27% were more than 35 years old. Regarding educational level, more than half of the women (54.8%) had a higher level of education, while 41.5% had only school education, and 3.95% were illiterate. The majority of participants were housewives (80.8%), followed by employed women (11.9%) and students (7.2%).

Table 2 presents the exposure to smoke among the women included in the study. About 33.7% of the women reported that their husbands currently smoked tobacco, of which 51% smoked indoors; 21% of the women reported not having smoking restrictions at home. In terms of workplace exposure, 11.5% of the women reported having experienced smoking in enclosed areas in their workplace in the past 30 days.

Table 3 shows the association between maternal factors and preterm births. SHS had a significant association with preterm birth (*p* = 0.014). This finding suggests that women exposed to SHS are more likely to experience preterm births. A significant relationship was also found between age and preterm birth (*p* = 0.039). Women older than 30 had a greater risk of preterm birth compared to younger women. A history of intrauterine fetal death was significantly associated with preterm birth (*p* = 0.044), indicating that women with such a history are more likely to experience preterm birth. Additionally, we found a highly significant relationship between the mode of delivery and preterm birth (*p* < 0.001); women who delivered via caesarean section were more likely to experience preterm birth.

Regarding education level, history of chronic disease, interval between pregnancies, and pregnancy-related infections, there were no statistical associations with preterm birth.

Table 4 presents the results of the multivariable logistic regression model for preterm birth. Exposure to SHS had a statistically significant association with preterm birth (*p* = 0.03), with mothers exposed to SHS being twice as likely to experience preterm birth. The odds ratio was 2.09, and the 95% confidence interval (CI) ranged from 1.06 to 4.13. This indicates that exposure to SHS poses a considerable risk of preterm birth.

## 4. Discussion

This analytical cross-sectional study examined the association between exposure to SHS and preterm labor in pregnant women in Al Madinah, Saudi Arabia. In the studied population, 28% of the women reported having been exposed to tobacco smoke during pregnancy, and the analysis indicated an association between SHS and preterm birth.

The WHO recognizes preterm birth as a primary contributor to neonatal mortality [7]. In the past ten years, the global incidence of preterm birth has risen, representing a significant public health issue [8].

The prevalence of preterm birth in our cohort was 11%, which aligns with a recent systematic review in Saudi Arabia that reported a prevalence of 7.89%. This is also consistent with global data indicating a prevalence of 9.9% [13].

Importantly, exposure to SHS emerged as a modifiable risk factor significantly associated with preterm birth. In our sample, 28% of the pregnant women indicated exposure to SHS. This is consistent with a previous national study conducted in Riyadh, which reported that 31% of pregnant women were exposed to SHS [14]. Both of these figures are slightly lower than that of the study conducted in Madinah in 2023, however, which reported that 46% of the pregnant women who participated were exposed to SHS [10]. This discrepancy could be due to self-reported data, which may lead to underreporting of smoking rates, especially in restrictive social environments where smoking carries a stigma.

Our findings indicate that 26.5% of the participants were exposed to cigarette smoke, corresponding with previous national studies from Riyadh. These previous studies documented cigarette exposure among pregnant women at rates of 31% and 24% [14,15].

Based on household smoking rules, 21% of the participants either permitted smoking or lacked any smoking-related rules in their homes, suggesting limited awareness of the harmful effects of passive smoke exposure. Our results also show that exposure to SHS is more prevalent at home than in the workplace, aligning with observations from various international studies [10,16,17,18].

We can attribute some of our findings to the extensive restrictions on smoking in public and workplace environments.

A significant association was observed between exposure to SHS and preterm birth (OR = 2.09; 95% CI: 1.06–4.13; *p* = 0.03). This finding is consistent with a previous study conducted in Riyadh, Saudi Arabia, which also reported that pregnant women exposed to SHS were two times more likely to experience preterm birth compared to those who were not exposed [19].

Worldwide findings corroborate these results. A recent case–control study conducted in Vietnam reported that 67% of mothers who delivered preterm infants were frequently exposed to SHS, compared to 51% of mothers who delivered at term [16]. Moreover, a cohort study in Shanghai reported that exposure to SHS significantly increased the risk of preterm birth compared to non-exposure (OR = 1.38; 95% CI: 1.05–1.81, *p* = 0.021) [20]. A systematic review and meta-analysis also revealed that exposure to SHS increased the risk of preterm birth [12].

The risk of SHS comes from the multitude of harmful chemicals found within cigarette smoke. These include nicotine and carbon monoxide, which are able to cross the placental barrier and come into direct contact with the developing fetus [12,20]. Nicotine inhibits prostacyclin synthesis, leading to vasoconstriction and subsequently reducing oxygen delivery to the fetus [21]. Meanwhile, carbon monoxide forms complexes with fetal hemoglobin, creating carboxyhemoglobin, which leads to significant diffusion impairments between maternal and placental blood [12]. This disruption can cause fetal hypoxia and increase the risk of preterm birth [22].

Many studies have indicated a significant association between preterm birth and exposure to SHS. However, specific results have varied; for example, studies from Portugal and Jordan have reported no statistically significant relationship between maternal smoking status and preterm birth [23,24].

Variations in the extent and intensity of exposure to SHS among pregnant women in different populations may account for this disparity. Supporting the dose–response concept, a recent study conducted in France in 2023 reported a significant increase in the risk of preterm birth when the husband was a heavy smoker—defined as smoking more than 20 cigarettes per day—compared to a light smoker [25].

The strong association between cesarean delivery and preterm birth in our study likely represents reverse causation, rather than cesarean delivery being a causal risk factor of preterm birth. In clinical practice, cesarean deliveries for preterm births are frequently medically indicated due to maternal or fetal complications such as pre-eclampsia, placental abruption, fetal distress, or breech presentation.

These results show the importance of advising pregnant women and promoting smoke-free homes. Physicians should frequently evaluate pregnant women for SHS exposure during antenatal visits. Additionally, it is crucial to educate spouses and other household members about the harmful effects of SHS. Household members who actively smoke should be motivated to stop smoking and provided with resources for smoking cessation.

This study has certain limitations. First, the sample was obtained from a single hospital in Madinah, which may limit the generalizability of the findings. Second, the assessment of SHS exposure relied on self-reported data, which could introduce recall bias and lead to underreporting. Third, the cross-sectional design has limitations in terms of establishing causality. Fourth, the lack of detailed smoking exposure quantification (including daily cigarette count) limits dose–response assessment. Finally, the inability to account for all potential confounding variables such as maternal stress and nutritional status may have influenced the observed associations.

We recommend undertaking more studies in Madinah to investigate and assess strategies designed to mitigate SHS exposure among pregnant women. These should focus on strategies for behavioral changes targeted at household members. Moreover, incorporating objective measurements of tobacco exposure would enhance the accuracy and reliability of the findings.

## 5. Conclusions

This study demonstrates that SHS exposure elevated the risk of preterm delivery among pregnant women in Madinah. The findings emphasize the essential need for preventive strategies to reduce SHS exposure in residential and public environments.

## Figures and Tables

**Table 1:** Demographical and past maternal characteristics of participants.

Characteristic	Frequency	Percent (%)
Age		
≤25	76	16.2
26–35	262	56.0
>35	130	27.8
Education level		
Illiterate	18	3.8
School education	194	41.4
Higher education	257	54.8
Employment status		
Employed	56	11.9
Housewife	379	80.8
Student	34	7.2
Have you had a previous difficult delivery?		
Yes	75	17.7
No	349	82.3
Have you previously experienced intrauterine fetal death?		
Yes	51	10.9
What is the average interval between pregnancies?	418	89.1
Less than 1 year	22	5.7
1 to 2 years	89	22.9
More than 2 years	278	71.5
Have you been diagnosed with any of the following chronic conditions?		
No chronic conditions	381	83.6
High blood sugar	30	6.6
Hypertension and heart diseases	2	0.4
Did you contract any infections during pregnancy?	56	11.9
Yes	63	13.4
No	406	86.6
Current delivery method		
Natural	233	49.7
Cesarean	236	50.3
Timing of delivery		
On time (9th month)	346	73.8
Preterm	52	11.1
Post-term	71	15.1

**Table 2:** Exposure to secondhand smoke, prevalence, and patterns among pregnant women.

Characteristics	Frequency	Percent (%)
Does your husband currently smoke cigarettes, shisha, or vape?		
Yes	158	33.7
No	297	63.3
I don’t know	14	3.0
If yes, what type of smoking does he do? (Among yes respondents)		
Cigarettes	37	26.5
Shisha	12	8.7
Vape	8	5.9
I don’t know	81	57.9
Does your husband smoke inside your home? (Among yes respondents)		
Yes	82	51.9
No	76	48.1
Which of the following best describes the smoking rules in your home?		
Allowed or no rules	100	21.3
Not allowed	369	78.7
How often did anyone smoke inside your home during your pregnancy?		
Daily	57	12.2
Weekly	10	2.1
Monthly	5	1.1
Never	348	74.2
I don’t know	49	10.4
In the past 30 days, has anyone smoked in enclosed areas at your workplace?		
Yes	54	11.5
No	292	62.3
I do not work outside the home	92	19.6
I don’t know	31	6.6

**Table 3:** Association between maternal characteristics and preterm delivery.

Characteristics		Preterm Delivery		*p* Value
		Yes	No	
		Frequency (%)	Frequency (%)	
Age	Age ≤ 30	15 (7.7%)	180 (92.3%)	0.039
	Age > 30	36 (13.8%)	224 (86.2%)	
Education level	Illiterate	3 (16.7%)	15 (83.3%)	0.753
	School education	21 (11.1%)	168 (88.9%)	
	High school and Master	27 (10.9%)	221 (89.1%)	
Have you previously experienced intrauterine fetal death?	Yes	10 (19.6%)	41 (80.4%)	0.044
	No	41 (10.1%)	363 (89.9%)	
Have you been diagnosed with any chronic conditions?	Yes	7 (9.5%)	67 (90.5%)	0.694
	No	41 (11.1%)	327 (88.9%)	
What was the average interval between pregnancies?	Less than 1 year	3 (14.3%)	18 (85.7%)	0.793
	1 to 2 years	9 (10.2%)	79 (89.8%)	
	More than 2 years	26 (9.7%)	243 (90.3%)	
Current delivery method	Cesarean	42 (18.3%)	187 (81.7%)	<0.001
	Natural	9 (4.0%)	217 (96.0%)	
Did you contract any infections during pregnancy?	Yes	4 (6.8%)	55 (93.2%)	0.248
	No	47 (11.9%)	349 (88.1%)	
Exposure to secondhand smoke	Yes	16 (18.8%)	69 (81.2%)	0.014
	No	35 (9.5%)	335 (90.5%)	

**Table 4:** Findings of multivariable logistic regression model for preterm birth.

Predictors	Comparison Groups	*p*-Value	Odds Ratio	95% CI
Secondhand smoke	Yes vs. No	0.03	2.09	1.06–4.13
Age	>30 years vs. <30 years	0.32	1.38	0.71–2.69
Education level	Higher education level versus lower education level	0.89	1.04	0.61–1.76
Have you previously experienced intrauterine fetal death?	Yes vs. No	0.02	2.62	1.13–6.03
Did you contact any infections during pregnancy?	Yes vs. No	0.27	0.54	0.18–1.61
Current delivery method	Normal vs. cesarian	<0.001	5.11	2.37–10.99

## Data Availability

All datasets generated and analyzed during this study are available from the corresponding author upon reasonable request.
